# Effects of short term elastic resistance training on muscle mass and strength in untrained older adults: a randomized clinical trial

**DOI:** 10.1186/s12877-015-0101-5

**Published:** 2015-08-12

**Authors:** Wagner Rodrigues Martins, Marisete Peralta Safons, Martim Bottaro, Juscelino Castro Blasczyk, Leonardo Rios Diniz, Romulo Maia Carlos Fonseca, Ana Clara Bonini-Rocha, Ricardo Jacó de Oliveira

**Affiliations:** 1 University of Brasilia, College of Physical Therapy, Campus Ceilândia, QNN 14, Ceilância Sul, DF 72220-140 Brazil; 2 University of Brasilia, College of Physical Education, Campus Universitário Darcy Ribeiro, Brasilia, DF 70904-970 Brazil; 3 Physical Education Department, Federal University of Pernambuco, Jornalista Anibal Fernandes Av, Campus Recife 50670-901, Recife, PE Brazil

**Keywords:** Randomized controlled trial, Resistance training, Muscle strength dynamometer, Dual-energy X-ray absorptiometry, Aging

## Abstract

**Background:**

The current recommendations on resistance training involving older adults have reported an improvement of body composition variables. Despite this, there is a lack of knowledge on how elastic resistance training (ERT) affects the muscle mass in older adults population. The purpose of this study was to determine the effects of a short-term ERT on muscle mass of health and untrained older adults.

**Methods:**

Forty older adults were randomized into two groups of 20 individuals each: Control Group (CG = 66.2 ± 6.6 years) and Training Group (TG = 69.1 ± 6.3 years). TG underwent an ERT twice a week during 8 weeks and control group did not receive any specific intervention. The primary outcome was the upper and lower limbs muscle mass, measured by Dual-energy x-ray absorptiometry. The secondary outcomes were knee isokinetic peak torque (PT) at 60°/s and 120°/s speeds and isometric handgrip strength. A 2×2 mixed model (group [TG and CG] × time [pre and post]) analysis of variance (ANOVA) was applied to determine the effect on primary and secondary outcomes.

**Results:**

The results of the ANOVA showed no significant effects in group x time interaction for (1) upper limbs fat free mass (F [1.38] = 1.80, *p* = 0.19, effect size [ES] = 0.1) and for (2) lower limbs fat free mass (F [1.38] = 0.03, *p* = 0.88, ES = 0.02). Regarding muscle strength, the ANOVA showed no significant effects in group x time interaction for (3) PT at 60°/s (F [1.38] = 0.33, *p* = 0.56, ES = 3.0), for (4) PT at 120°/s (F [1.38] = 0.80, *p* = 0.38, ES = 4.1) and for handgrip strength (F [1.38] = 0.65, *p* = 0.42-value, ES = 0.9). Analysis of PT in TG showed a significant change of 4.5 %, but only at 120°/s (*p* = 0.01) when comparing pre and post-training (time interaction).

**Conclusions:**

Eight weeks of ERT did not show significant changes in muscle mass and strength of untrained older adults.

**Trial registration:**

NCT02253615 (09/25/14)

## Background

Aging is a complex process characterized by structural and physiological changes in various systems of the human body that can compromise anthropometric (e.g. muscle mass, regional adiposity) and neurovascular (muscle strength, motor control) variables, and reduce the physical independence of the older adults [1]. Decreased muscle strength is considered an important physiological change related to aging. It is involved in the pathogenesis of frailty and disability that leads to decrease instrumental activities of daily living [2–5], increasing the risk of fall, morbidity and mortality [6, 7]. Changes in body composition (percentages of fat, bone, water and muscles), e.g., measured by dual-energy X-ray Absorptiometry, is another feature of aging that can affect the health of the older adults; as well as body fat increase (visceral depots) and loss of muscle mass (sarcopenia) is related to metabolic and cardiovascular disease risks due to the relationship with glucose tolerance, hyperinsulinemia, hypertriglyceridemia and arterial hypertension [1].

The current recommendations on resistance training programs involving older adults have reported an improvement of muscle function and body composition variables. These studies show that systematic use of weight resistance devices has improved muscle strength and power, functional skills and muscle mass [1, 8–13]. Unfortunately, some untrained elderly with joint dysfunction or in physical therapy treatment may not lift the necessary weight to produce positive muscle adaptation, due to pain experience and or decrease in motor control (dynamic instability) [14]. Although this type of training with free-weights or weight machines typically found in gyms and health clubs are common among young adults, older adults may not have as easy access to this equipment due to lack of facilities or financial resources [15].

However, elastic resistance training may be more easily available and affordable exercise for older adults populations [16, 17]. These elastic devices (bands and tubes) are practical and low cost [18]. Training with elastic resistance has been increasingly used because it allows functional movement patterns, more versatile and accessible for individuals of different ages and is more readily available in a variety of clinical conditions. One of its most versatile characteristic is the portability [19–26] that allows training programs in outdoors situations. In addition, overload training stimulus could be self-regulated by the use of the color-code (elastic tubes with different dimensions/forces) and corresponding target rating perceived exertion (e.g., extremely easy [0 numerical responses] to extremely hard [10 numerical responses]). Regarding the effects of elastic resistance training, Colado et al. [17] demonstrated, in postmenopausal females, similar outcomes (functional capacity, vertical jump, isometric strength) when compared with traditional machine weight exercise programs.

Most of the studies on the effects of elastic resistance training in older adults assessed short (i.e. 12 weeks) and medium (i.e. 12 – 24 weeks) term interventions on muscle strength (isotonic elastic resistance training), and have shown positive outcomes in isometric, isotonic and isokinetic muscle strength [27–32]. The first studies on this topic applied medium-term programs to demonstrate the effectiveness of elastic resistance training on muscle strength, and more recent studies have already demonstrated muscle strength increase with short-term programs [17, 28, 31, 33]. The first studies investigating this relationship recruited postmenopausal middle-aged women [14, 33] (active [14]; healthy and living independently in the community [28]), and middle-aged obese women with type 2 diabetes (patients who visiting the Hospital) [34]. There is a lack of knowledge, however, on how elastic resistance training affects the muscle mass in upper and lower body in older adults. Therefore, it is important to know if the short-term training program, as a specific training variable, could improve muscle mass in untrained older adults.

The purpose of this study was to determine the effects of a short-term training with elastic resistance on muscle mass of health and untrained older adults, and assess if eight weeks of training are sufficient to change upper and lower limbs’ muscle strength.

## Methods

### Study design

Parallel randomized trial of two groups.

### Participants

Convenience sample of 40 healthy and untrained older adults (12 males and 28 females) with a minimum of 60 years of age were advertised in newspapers and posters. The posters were also distributed at the Centers for Integrated Health of Older Adults in Brasilia, Brazil. All the participants recruited live in Brasilia and each presented a medical certificate of suitability or fitness to practice resistance training. The exclusion criteria were subjects suffering from orthopedic, neurologic, rheumatologic, metabolic or heart conditions, and uncontrolled arterial hypertension. Additional exclusion criteria included undergoing estrogen therapy, use of cardiac pacemaker, knee or hip arthroplasty, the presence of fragments from previous osteosynthesis; orthopedic surgery, fractures, muscle injury or the practice of resistance training within the last 6 months, and having a Short Physical Performance Battery (SPPB) score of ≤ 9.

Among 84 subjects expressing interest, 20 (24 %) were eliminated because of the exclusion criteria: heart disease (*n* = 3), current muscle training (*n* = 1), inguinal hernia surgery (*n* = 1), fibromyalgia (*n* = 1), diabetes (*n* = 6), cancer (*n* = 1), heart surgery (*n* = 1), cerebrovascular accident (*n* = 1), rheumatoid arthritis (*n* = 1), peripheral neuropathy (*n* = 1), sequelae of poliomyelitis (*n* = 1), congestive heart failure (*n* = 1) and severe shoulder disability (*n* = 1). Another 17 subjects (20 %) lost interest after learning about the research objectives.

**Fig. 1:**
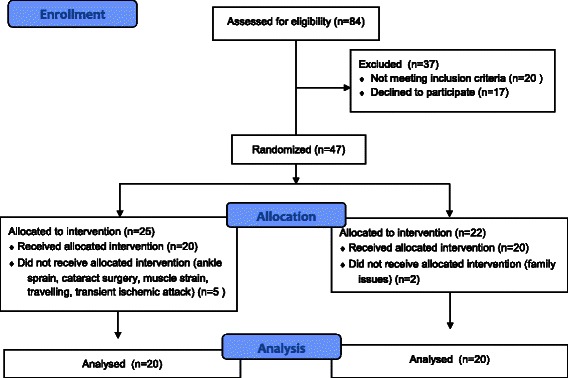
Flow diagram of the process trough the phases of the study

Forty-seven subjects (55.9 %) signed the consent form and were randomly allocated in two groups through randomized block technique: 25 in the training group (TG) and 22 in the control group (CG). Block randomization is a commonly used technique in clinical trials, designed to reduce bias and achieve balance in the allocation of participants to treatment arms [35]. After participants selection, a randomization was performed using a computerized random number generator (Excel, Microsoft Office 2008) through every blocks (two individual) for sequence generation (two groups). A sequentially numbered, opaque, sealed assignment envelope was opened by a researcher in front of the participant; each envelope contains a concealed allocation number. The same researcher who generated the random allocation sequence performed the enrollment of participants. Five subjects from the TG and two from the CG dropped the study, leaving each group with 20 subjects. All the subjects in both groups underwent the study between March and November 2013. This research project and the informed consent were approved by the Ethics Committee of the Faculty of Health Sciences – Brasília University on July 21^st^, 2011 under the registration number 081/11. The informed consent was obtained from all participants after the procedure of study selection criteria. For publication of images (Fig. 1) was obtained the consent of the participant during the experiment.

### Primary outcomes assessment

The primary outcome was the muscle mass assessment. Whole body and regional composition were assessed using a fan-beam dual-energy X-ray Absorptiometry (DEXA) (GE Electric Company®, Lunar Prodigy). Regional analysis of Arms (upper limbs) and Legs (lower limbs) were assessed according to anatomical landmarks by the same technician using computer algorithms provided by enCORE*®* software. The coefficient of variation (CV) obtained for measurements performed at the laboratory of the University of Brasilia was 0.9 and 1.9 % for body fat and lean body mass, respectively. These measurements aimed to establish the reliability of DEXA protocol in previous study [36].

### Secondary outcomes assessment

The secondary outcome is the muscle strength assessment. Lower limb muscle strength was assessed with an isokinetic dynamometer (Biodex System® III, Shirley) and upper limb muscle strength with an isometric dynamometer (Jamar®, Hand Dynamometer).

Isokinetic muscle strength of the lower limbs was measured through peak torque (PT) during concentric extension of the dominant knee at 60°/s and 120°/s speed, according to an adapted protocol developed by M Bottaro, AF Russo and RJ de Oliveira [37]. To define the dominant side the subjects were asked with which leg they would kick a ball [38]. Pre and post-training protocol was performed in the following order: 1st – a warming of 10 repetitions of the dominant knee extension at 300°/s of speed; 2nd – 3 sets of 4 repetitions at 60°/s; 3rd – 3 sets of 4 repetitions at 120°/s. Between the series of the 2^nd^ and 3^rd^ exercises 1-min interval was used, and between the 2^nd^ and 3^rd^ exercises the subjects rested for 2 min. At this point, the belts fixating a subject’s trunk were loosened to alleviate any discomfort. Speed of each exercise was set at 60 and 120 °/s based on research indicating most daily activities of older adults involve low speed muscle work [38].

In order to minimize the movement artifacts, the subjects were asked to sit on the dynamometer chair in a comfortable position and seat belts were used to stabilize trunk, hips and thighs. To align knee rotation axis with the dynamometer rotation axis, lateral epicondyle of the femur was set as the bone reference. Sitting position of subjects allowed free and comfortable knee flexion and extension, with a range of motion set at 80° measured from the maximum knee flexion.

Chair height and position, backrest adjustment, dynamometer position and resistance arm adjustment were recorded to standardize each subject’s individual position. Gravity correction was made by measuring resistance arm torque and subject’s leg at full knee extension. During the test, all subjects kept their arms crossed, holding the belts at shoulder height. Verbal stimuli and Biodex visual feedback were used to promote maximum effort. The verbal stimulus was standardized by the same investigator. The highest PT at each speed (pre and post-intervention) was recorded for statistical analysis. The reliability of isokinetic peak torque test was assessed in a test-retest protocol (pre-experimental study). For relative reliability, we found intraclass correlation coefficients (ICC) of 0.94 (60°/s) and 0.96 ate (120°/s). For absolute reliability, we obtained a systematic bias of 1.4 Nm (60°/s) and 0.8 Nm (120°/s), and a random error of 15.1 Nm (60°/s) and 12.1 Nm (120°/s).

Isometric handgrip was assessed on the dominant side, which was defined as the preferred hand for writing, eating and carrying heavy objects [39]. During handgrip assessment, subjects were seated, dominant shoulder in rest position; elbow flexed 90° without support, forearm and wrist at neutral position. Dynamometer’s anterior grip was placed in the second position of contact when each subject performed 3 maximum isometric contractions of five seconds with one-minute intervals between contractions. Verbal stimuli were used to promote maximum effort. The highest handgrip strength at three sets (pre and post-intervention) was recorded for statistical analysis.

### Procedures

The dependent variables were measured in the same order in pre and post-training, in the following sequence: 1st – anthropometry; 2nd – DXA; 3rd – isometric dynamometry; and 4th – isokinetic dynamometry. The first two weeks of the study were dedicated to pre-training assessment (separate from the training). After the end of training sessions, post-training assessment was conducted respecting at least a two-day interval between the last session and the tests, and all measurements were obtained in the morning (between 8 and 11 a.m.) by the same examiner, who was blinded for individual/groups.

The participants were allocated in 2 groups: Training Group (TG) (*n* = 20; 14 females; 6 males) and Control Group (CG) (*n* = 20; 14 females; 6 males). The subjects underwent a two-week familiarization period (detailed below) [40–42], and then an eight-week strength-training regimen performed twice a week. The eight-week training consisted of four exercises for lower limbs and three exercises for upper limbs, which were alternately performed with a two-minute interval between them during familiarization and training periods. Lower limb exercises consisted of (1) hip flexion (unilateral exercise performed in standing), (2) hip extension (unilateral exercise performed in standing), (3) knee flexion (unilateral exercise performed in sitting) and (4) Knee extension (unilateral exercise performed in sitting). Upper limb exercises consisted of (1) bench press (bilateral exercise performed in standing), (2) rowing and (3) high pulley exercise (bilateral exercise performed in standing). Control group did not receive any specific training or placebo condition. Figure 1 shows the exercises employed in experimental group.

In order to determine the intensity of the exercises for all participants, the method described in the study of JC Colado and NT Triplett [33] was used, which monitors the target number of repetitions (TNR) and the rate of perceived exertion (RPE) by OMNI-RES scale [43]. The use of OMNI-RES was standardized according Colado et al. [33]. The participants trained this method during two weeks of familiarization (four sessions), which occurred with TNR of 15 repetitions (two sets) and low intensity exercises of OMNI-RES (1 to 4 in scale) for all exercises. The TNR of 15 repetitions were maintained for the eight-week resistance training, with two sets during the first four weeks and three sets for the last four weeks. Regarding the RPE, during the first four weeks, OMNI-RES was set between 5-7 and 8-10 for last four weeks; thus, two target OMNI RES ranges to intensity progression of exercises were individually prescribed.

To increase the RPE a progression color-scale (Elastos®, elastic tubes) was used according to mechanical evaluation of the device in laboratory (Martins [44]). This is a color-based scale with 7 levels of resistance represented by the colors: Yellow, Red, Green, Blue, Black, Purple and Gold (weakest to strongest). Intensity was then adjusted by changing from the current tube to the next color presented in the scale (e.g. from Yellow to Red tube). When a subject reached the level of resistance Gold, another Elastic tube was added while always respecting the progression scale, i.e., Gold plus Yellow. As the tube length variations (percentage of elongation) can affect exercise intensity, an adhesive tape on the ground (reference) showed the maximum elongation for all exercise to control tube elongation. The exercise was then standardized and performed at 100 % (knee extension; bench press; rowing), 150 % (knee flexion; knee extension), or 200 % (hip extension; high pulley) of tube elongation.

Training was conducted in the Laboratory of Ergonomics and Bodybuilding at the Olympic Center of the University of Brasília. Before entering the laboratory, subjects performed a 5-min walk warm-up on the outdoor sports court followed by instruction and training on proper stretching exercises. On the first day of assessment tools, all subjects (TG and CG) were instructed not to engage in any physical activity, nor change their daily activities or eating habits. They were informed of possible adverse reactions and were supervised by both a physiotherapist and a physical education teacher during all training sessions.

### Statistical analysis

The sample size was calculated considering: (1) the two-way analysis of variance (repeated measures, within-between interaction); (2) two groups; (3) type I error = 5 %; (4) type II error = 20 %; (5) the power of the statistical test = 80 %; (6) effect size = 0.20. With this parameters, G*Power software (version 3.1.9.2) calculated a total sample size of 52 individuals (26 per group).

Mean and standard deviation (SD) were used for descriptive statistics. Shapiro-Wilk’s test was used to test data normality and Bartlett’s test for homoscedasticity. Considering these assumptions, every dependent variable was analyzed by 2×2 mixed model (group [TG and CG] x time [pre and post]) analysis of variance (ANOVA), using Bonferroni Post-hoc method when a statistically significant difference was found. Between-group effect sizes (ES) were calculated as follows: ES = M_E_ – M_C_/SD_C_; where M_E_ = the mean of the experimental group, M_C_ = the mean of control group, and SD_C_ = the standard deviation of control group.

A student *T* Test was used to verify the presence of statistical difference between groups, relating anthropometric variables (age, weight, height, body mass index [BMI], and the International Physical Activity Questionnaire [IPAQ] score) during the pre-training period. Data were analyzed using Prism 6 software, and a significance level of *p* ≤ 0.05 was adopted to all variables.

## Results

**Fig. 2:**
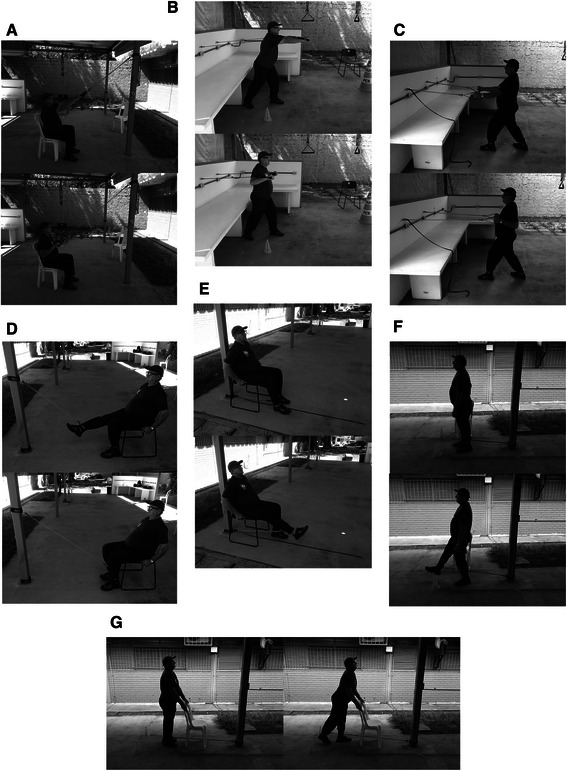
Exercises in elastic resistance training program. high pulley, (**b**) bench press, (**c**) rowing, (**d**) knee flexion, (**e**) knee extension, (**f**) hip flexion, (**g**) hip extension

Figure 2 shows the flow diagram of the process trough the phases of study. The reasons to drop out of the study (*n* = 7) were transient ischemic attack (*n* = 1; CG), ankle sprain (*n* = 1; EG), cataract surgery (*n* = 1; EG), muscle strain (*n* = 1; EG), family issues (*n* = 2; CG) and travelling (*n* = 1; EG). Nine subjects (45 %) from TG reported adverse events: lateral epicondylitis (*n* = 1), plantar fasciitis (*n* = 1), low back pain (*n* = 2), lumbosciatalgia (*n* = 1), Knee pain (*n* = 1), hypertension (*n* = 1) and muscle pain (*n* = 2). These nine subjects were assessed as to if their clinical condition would implicate in decrease of motor control during the exercises. These conditions did not compromise the training and testing procedures.

Demographic characteristics are presented in Table 1. There were no statistical differences between groups at pre-training phase for age (*p* = 0.16), weight (*p* = 0.19), height (*p* = 0.31), BMI (*p* = 0.46) and IPAQ (*p* = 0.47). Descriptive data for outcome measures are shown in Table 2. Both male and female were analyzed together because no statistical differences between groups were found for all outcomes.

**Table 1:** Baseline physical characteristics. Data are presented as the mean (± SD)

	Control group	Training group
Subjects (n)	20	20
Age (years)	66.2 (6.6)	69.1 (6.3)
Height (cm)	1.64 (0.10)	1.61 (0.09)
Body mass	72.7 (11.7)	68.6 (7.8)
BMI (Kg/m^2^)	27.5 (2.5)	26.8 (3.2)
SPPB	10.4 (1.0)	10.5 (1.0)
IPAQ (RMR-min/week)	4657 (4584)	3491 (4300)

*SPPB* Short Physical Performance Battery; *RMR-min* resting metabolic rate-minutes per week

**Table 2:** Primary and secondary outcomes at baseline and post-training. Data are presented as the mean (± SD)

Variables	Group	Mean		Effect			
		Baseline	Post	Δ%	Effect size between group	Time	Group × Time
ULFFM (g)	TG	4.2 (1.1)	4.4 (1.0)	4.7	0.1	0.28	0.19
	CG	4.9 (1.3)	4.9 (8.9)	0.0			
LLFFM (g)	TG	12.6 (2.7)	12.5 (2.6)	0.0	0.03	0.92	0.88
	CG	13.5 (4.0)	13.5 (3.8)	0.0			
Handgrip (Kgf)	TG	32.8 (9.5)	33.7 (9.1)	2.9	0.9	0.12	0.42
	CG	35.1 (10.7)	35.4 (10.8)	0.8			
PT 60°/s (N.m)	TG	118.5 (41.0)	121.6 (39.7)	2.6	3.0	0.29	0.56
	CG	124.8 (38.3)	125.7 (36.9)	0.7			
PT 120°/s (N.m)	TG	92.7 (27.9)	96.9 (29.7)	4.5	4.1	0.01^a^	0.38
	CG	100.3 (32.0)	102.3 (32.0)	2.0			

*PT* peak torque; *ULFFM* upper limb fat-free mass; *LLFFM* lower limb fat-free mass

^a^ intra-group difference (post – pre) to TG (*p* = 0,01)

### Primary outcomes

There was no significant difference between groups for limb fat-free mass (LFFM) of upper limbs (*p* = 0.35) and lower limbs (*p* = 0.87) at the pre-intervention moment. The results of the ANOVA showed (1) no significant effects (F [1.38] = 1.80, p = 0.19, ES = 0.1) in group x time interaction for upper limbs fat free mass (ULFFM) and no significant effects (F [1.38] = 0.03, *p* = 0.88, ES 0.02) in group x time interaction for lower limbs fat free mass (LLFFM).

### Secondary outcomes

There was no significant difference between groups for PT at 60°/s (*p* = 0.56), PT at 120°/s (*p* = 0.37) and handgrip (*p* = 0.42) at the pre-intervention moment. ANOVA showed (1) no significant effects (F [1.38] = 0.33, *p* = 0.56, ES = 3.0) in group x time interaction for PT at 60°/s, (2) no significant effects (F [1.38] = 0.80, *p* = 0.38, ES = 4.1) in group x time interaction for PT at 120°/s and no significant effects (F [1.38] = 0.65, *p* = 0.42-value, ES = 0.9) in group x time interaction for handgrip strength. Post-hoc analysis of PT in TG showed significant change at 120°/s (4.5 %), when comparing pre and post-training (time; *p* = 0.01).

## Discussion

The purpose of this study was to determine the effects of a short-term training with elastic resistance on muscle mass of healthy and untrained older adults. The results suggest no effects of the 8-week training program for upper and lower body muscle mass in untrained older adults.

The first systematic review to analyze treatment affects for LBM across multiple training dosages and potential mediating variables was the study MD Peterson, A Sen and PM Gordon [12]. The pooled estimate of LBM change from baseline to post intervention (81 treatment cohorts; 49 studies) was 1.1 Kg (95 % CI = 0.9-1.2 Kg; *p* < 0.001). By using multiple meta-regression, a strong association was determined between the volume of training and the magnitude of the LBM change, with higher volume (total number of sets performed per session) interventions being associated with greater LBM increase. Volume ranged from 7 to 39 total sets per session and the majority of included studies conformed to the American College of Sports Medicine recommendations (i.e., 8-10 exercises for one to two sets of full-body exercises). Regarding the volume of training as a predictor of LBM, the present study performed 14 and 21 total sets on first and second four-week period, respectively. These two values are within the range of volume training identified by MD Peterson, A Sen and PM Gordon [12] as a predictor of changes in LBM, but no significant effects on muscle mass in the group x time interaction were show after resistance training. Although the present study has employed a good volume of training, the physiological adaptations on muscle mass are also respond to intensity mode.

Regarding the intensity mode, to produce muscle hypertrophy, the American College of Sport’s Medicine also recommends exercising at ≥70 % of one’s concentric 1-repetitum maximum (1RM) [1], but the difficulty lies on setting the elastic band force relative to 1RM to meet the recommendations and produce muscle hypertrophy. Currently, 1 RM tests are not validated for elastic resistance training, because most of the available devices do not have a determined load scale for exercises, and, although there are several trials to determine elastic force, none of them developed a valid and reliable method for elastic tubes and bands for scientific and professional use [44]. Thus, in the present study the 1RM test was not employed.

Despite this technical limitation for using elastic resistance, JC Colado and NT Triplett [33] developed a successful model of elastic band exercises to improving strength (measured by functional capacity; 28 % of increase measured by squat test; 30 % of increase measured by knee push-up test), decrease in fat mass and increase in fat-free mass (measured by 8-polar bioelectrical impedance analyzer; increase of 1.2 % on fat-free mass [FFM]; decrease of 1.7 % in fat mass [FM]) for sedentary middle-aged postmenopausal women (elastic band group with 54.14 [±2.87] mean age), who had exercise intensity equalized by monitoring the TNR and RPE in active muscles. The program lasted 10 weeks, twice a week. Six exercises (major muscle groups of the whole body) with 20 repetitions were performed at an intensity of 5 in OMNI-RES scale for the first 4 weeks, and 7 for the next 6 weeks. In the first 4 weeks, 2 sets were performed for the lower and 1 set for the upper extremities; from weeks 5 to 8, they were equalized for the upper and lower body; and for weeks 9 and 10, the number of sets was 3.

JC Colado, X Garcia-Masso, ME Rogers, V Tella, J Benavent and EH Dantas [15], in another short-term study, analyzed the effects of a supervised strength training program using three devices (Weight machines, Elastic bands, and Aquatic devices that increase drag force) on body composition and physical capacity (knee push-up test, squat test and crunch test) in postmenopausal women (elastic band group with 54.14 [±0.63] years mean age). The periodized training program in elastic bands group was the same (TNR + RPE) by JC Colado and NT Triplett [33] and the only difference was an active 30-s recovery period of gentle jogging between exercises. There was a decrease in FM of 1.93 % and a 1.15 % increase in FFM (significant difference between pre and post test). In addition, the elastic bands group showed a significant increase in the post-test of 30.62 %, 16.27 % and 27.4 % in the number of push ups, crunches and squats respectively (difference between pre and post test).

Our results agree with the study RS Thiebaud, JP Loenneke, CA Fahs, LM Rossow, D Kim, T Abe, MA Anderson, KC Young, DA Bemben and MG Bemben [14], in which postmenopausal women (61 ± 5 years) were assigned to a moderate-to-high-intensity elastic band group (*n* = 8; three sets of ten repetitions) or a low-intensity elastic band group combined with blood flow restriction (*n* = 6; one set of 30 repetitions followed by two sets of 15 repetitions for upper body exercises under blood flow restriction; and lower body without blood flow restriction). Each group performed seated chest press, seated row and seated shoulder press with elastic bands three times a week for eight weeks. Elastic bands colors progressed in each group by having participants maintain a rate of 7–9 on the OMNI scale throughout training (i.e. TNR + RPE). The DEXA values revealed no differences in time and group x time interactions for upper and lower body muscle mass. However, muscle strength (1RM test in machines) significantly increased for the chest press, seated row and shoulder press, but no differences were found between groups (only intra groups differences).

The use of TNR and RPE in randomized clinical trials appears to be effective in determining the intensity of the elastic band training, once favorable outcomes (functional capacity; FM; FFM; 1RM) were obtained in short-term resistance training [14, 15, 33]. Moreover, the use of OMNI-RES have already demonstrated sensitivity in differentially measuring PRE from active muscle groups, as well as the total body (i.e. total weight lifted) over increased loads in separate sets of upper and lower body isotonic exercises [43]. The concurrent validity of the OMNI-RES was also done for elastic bands during isotonic resistance exercises [45].

In general, studies found muscle hypertrophy after resistance training with weight machines, and there is evidence that the significant muscle mass improvement starts after 9 weeks of training [46]. Besides training length, muscle hypertrophy seems to depend on muscle fiber recruitment patterns, i.e., when high load training is performed the type II fibers have a greater potential for muscle growth and a higher force area per unit than type I fibers [11]. MA Fiatarone, EC Marks, ND Ryan, CN Meredith, LA Lipsitz and WJ Evans [47] proved this 20 years ago after demonstrating the effects of a short-term training (high intensity) on muscle hypertrophy in subjects over 90 years of age. At the same time, WR Frontera, CN Meredith, KP O’Reilly, HG Knuttgen and WJ Evans [48] also observed that a 12-week high intensity training did increase muscle mass when analyzed by CT scan and muscle biopsy.

Besides the evidence underlying the improvement on muscle mass (association between volume, intensity and duration of training) another specific training variables need to be addressed to provide benefits on muscle mass in older adults. Recently, circuit training, a form of strength training with repeated sequence of exercises with short rest periods (35 s) other than those used in typical resistance training (2-3 min), was found to be effective at lowering body fat, increasing muscle mass, and improving functional capacity and strength [49]. This circuit training has also been shown to be more effective at lowering body fat mass than traditional strength training in aging population [50].

Regarding the muscle function, WJ Kraemer, SJ Fleck and WJ Evans [51], TR Henwood and DR Taaffe [52] suggested that short-term training of varied training protocols would improve muscle neural activity, motor unit synchronization and an increase in agonist muscles activation; and these changes would improve muscle strength. Our results showed an increase statistically significant only in the isokinetic PT at 120°/s of TG. Despite these results are a sign of muscle strength improvement, it does not support an evidence of training effect, because no difference in CG was showed. DE Krebs, DM Scarborough and CA McGibbon [30] also did not observe significant changes in their six-week elastic resistance training. They observed 3.7 % of improvement on isometric knee extension in older adults with functional disability (TG, *n* = 6), and no statistical difference to control group. Their protocol had 11 exercises with diagonal and rotational movements to mimic daily activities, performed three times a day. They used the progression color-scale to control training intensity and they increased the intensity when a subject performed one set of 10 repetitions without fatigue or loss in quality of movement (the authors did not describe how they controlled fatigue and quality of movement). They attributed the results to the small sample and the lack of a high intensity overload during training. TM Damush and JG Damush, Jr. [27] observed positive outcomes in maximum dynamic force of major muscle groups and negative outcomes in isometric handgrip after eight weeks of resistance training (elastic bands exercises, seven exercises, twice a week) in 62 healthy older adults (TG, *n* = 33; CG, *n* = 29), and they used Borg scale (rating of perceived exertion) to control the intensity. They found an increase in force in the TG compared with CG as follows: 15 % increase in *latissimus dorsi* muscle; 13 % increase in *pectoralis major* muscle and 25 % in *quadriceps femoris* muscle. F Ribeiro, F Teixeira, G Brochado and J Oliveira [28] found a positive association between elastic resistance training and ankle isometric force of institutionalized older adults (TG *n* = 24; CG *n* = 24) after a six-week protocol of ankle flexion and extension (3 × 10 repetitions), three times a week, controlling intensity with a progression color-scale. They observed 4.2 Kg (50 %) isometric force increase in ankle flexion and 4.5 Kg (34 %) increase in ankle extension with statistical significance. Only these three studies investigated the effects of short-term resistance training on dynamic muscle strength of older adults.

With respect to isometric handgrip strength, JA Serra-Rexach, N Bustamante-Ara, M Hierro Villaran, P Gonzalez Gil, MJ Sanz Ibanez, N Blanco Sanz, V Ortega Santamaria, N Gutierrez Sanz, AB Marin Prada, C Gallardo, et al. [53], as well as TM Damush and JG Damush, Jr. [27], did not observe any statistical difference between groups (experimental vs. control group) in an eight-week elastic resistance training program with subjects between 90-96 years old (*n* = 20). NW Cheung, N Cinnadaio, M Russo and S Marek [21] applied a 16-week protocol (biceps and triceps brachialis exercises – 3 × 10 repetitions) and did not observe any statistical difference between TR and CG. These negative outcomes could relate to the exercise type and muscle action. According to L Griffin, PE Painter, A Wadhwa and WW Spirduso [54] and CA Speed and R Campbell [55], it is necessary to perform isometric exercises in short and medium-term (four and twelve weeks respectively) to change handgrip significantly. In this study, we used only multi-articular isotonic exercises for the whole body; the exercises employed did not have, thus, the type of contraction required to produce isometric grip strength.

The lack of significant changes in muscle mass and strength in the present study may have occurred partially because of the too short-term period associated with the fact that the resistance applied in elastic training is, thus, essentially subjective (RPE). The present study applied a similar method by adjusting the TNR and the color of the elastic tube according to OMNI RES scale for the participants, but the exact intensity cannot be calculated, only merely estimated. Until now, several studies had found great difficulty in controlling the intensity of exercises performed with elastic bands [15], so this could be considered as a limitation of the present study. Furthermore, to ensure appropriate overload training stimulus in future studies, a pre-experimental orientation and practice involving color-code and corresponding OMNI RPE could help subjects learning how to select an appropriate target elastic tension (i.e. target the correct intensity). In the present study this practice was employed only in the two weeks of familiarization with the exercise program (fours sessions).

In addition, changes in muscle strength after resistance training are assessed using a variety of methods, including isometric, 1RM, and multiples repetition (e.g., 3-RM) maximum-effort protocols. In general, strength increase seems to be greater with measures of 1-RM or 3-RM performance compared with isometric or isokinetic measures (Chodzko-Zajko [1]). We assessed muscle strength only with isometric and isokinetic tests, and our results could be influenced by the methods of measurement (i.e. could be sub estimated). It is important, however, that future studies consider this.

Another point is the choice of multi-articular exercises, because those exercises demand neural coordination between muscles of several muscle groups; also, they require a proper static balance and continuous body posture adjustment, which requires more learning sessions (longer periods) than mono-articular exercises sessions. We presumed that the two-week familiarization period would assure neuromuscular adaptation (static and dynamic postural control adjustments), but the observation of some wobbling and motor dyscoordination at the start of the training undermined this presumption and could have affected the results. The demand for balance and motor coordination may have inhibited the production of strength and muscle endurance.

Strategies to prevent or treat muscle mass loss related to age have received much attention, yet we could not find studies on short, medium or long-term elastic resistance training of older adults. It is important, therefore, to know if short-term elastic resistance training has an adequate duration (i.e. in terms of specific training variable) to provide minimum and significant benefits for muscle mass in untrained older adults. To our knowledge, this is the first study to analyze the effects of a short-term training with elastic resistance on muscle mass of health and untrained older adults population.

## Conclusions

The present protocol failed to show significant changes in muscle mass and muscle strength after eight weeks of elastic resistance training of untrained older adults. Thus, the main issue involving the relationship between the variables of short-term training with elastic resistance, muscle structure and muscle function has yet to be resolved. Further studies need to be conducted considering the limitations presented in this study.
